# Perspectives on interferon-alpha in the treatment of polycythemia vera and related myeloproliferative neoplasms: minimal residual disease and cure?

**DOI:** 10.1007/s00281-018-0700-2

**Published:** 2018-09-10

**Authors:** Hans Carl Hasselbalch, Morten Orebo Holmström

**Affiliations:** 1 Department of Hematology, Zealand University Hospital, Sygehusvej 10, 4000 Roskilde, Denmark; 2 Center for Cancer Immune Therapy, Department of Hematology, Herlev Hospital, Herlev, Denmark

**Keywords:** Pegylated interferon-alpha2, Myeloproliferative neoplasms, MPNs, MPN, Inflammation, Combination therapy, Ruxolitinib, DNA-hypomethylator, Statins, Minimal residual disease, MRD, Cure, Vaccination strategies

## Abstract

The first clinical trials of the safety and efficacy of interferon-alpha2 (IFN-alpha2) were performed about 30 years ago. Since then, several single-arm studies have convincingly demonstrated that IFN-alpha2 is a highly potent anti-cancer agent in several cancer types but unfortunately not being explored sufficiently due to a high toxicity profile when using non-pegylated IFN-alpha2 or high dosages or due to competitive drugs, that for clinicians at first glance might look more attractive. Within the hematological malignancies, IFN-alpha2 has only recently been revived in patients with the Philadelphia-negative myeloproliferative neoplasms—essential thrombocytosis, polycythemia vera, and myelofibrosis (MPNs)—and in patients with chronic myelogenous leukemia (CML) in combination with tyrosine kinase inhibitors. In this review, we tell the IFN story in MPNs from the very beginning in the 1980s up to 2018 and describe the perspectives for IFN-alpha2 treatment of MPNs in the future. The mechanisms of actions are discussed and the impact of chronic inflammation as the driving force for clonal expansion and disease progression in MPNs is discussed in the context of combination therapies with potent anti-inflammatory agents, such as the JAK1–2 inhibitors (licensed only ruxolitinib) and statins as well. Interferon-alpha2 being the cornerstone treatment in MPNs and having the potential of inducing minimal residual disease (MRD) with normalization of the bone marrow and low-*JAK2*V617F allele burden, we believe that combination therapy with ruxolitinib may be even more efficacious and hopefully revert disease progression in many more patients to enter the path towards MRD. In patients with advanced and transforming disease towards leukemic transformation or having transformed to acute myeloid leukemia, “triple therapy” is proposed as a novel treatment modality to be tested in clinical trials combining IFN-alpha2, DNA-hypomethylator, and ruxolitinib. The rationale for this “triple therapy” is given, including the fact that even in AML, IFN-alpha2 as monotherapy may revert disease progression. We envisage a new and bright future with many more patients with MPNs obtaining MRD on the above therapies. From this stage—and even before—vaccination strategies may open a new horizon with cure being the goal for some patients.

## Introduction

About 60 years ago, interferon (IFN) was discovered by Isaacs and Lindenmann [1] who described this cytokine to be able to interfere with virus replication. Later, the IFN receptor was identified and shortly after the JAK/STAT-signal transduction pathway as described in several recent reviews [2–6]. It early became apparent that one of the mechanisms of action of IFN-alpha2 involved stimulation of immune cells [7, 8]. Due to all the other properties of IFN, including its antiproliferative, immunomodulatory, and antiangiogenic effects, great interest in the potential use of IFN in the treatment of several malignancies was soon raised. The production and purification of human leukocyte IFNs [9] were followed by the first clinical study in the late 1970s on the efficacy of IFN-alpha2 in multiple myeloma (MM) [10]. Soon after, IFN-alpha2 was cloned, allowing large amounts of IFNs to be produced for experimental research and clinical trials, opening an exciting era of several years, in which the safety and efficacy of IFN was tested in a variety of hematological malignancies. Among these are multiple myeloma, hairy-cell leukemia (HCL), chronic myelogenous leukemia (CML), the classical Philadelphia-negative chronic myeloproliferative neoplasms and essential thrombocythemia (ET), polycythemia vera (PV) and primary myelofibrosis (PMF) (MPNs), the hypereosinophilic syndromes, and systemic mastocytosis (SM). Outstanding breakthroughs in the treatment of HCL and CML with IFN-alpha2 were confirmed in several large clinical trials. Thus, a large proportion of patients with HCL achieved long-lasting complete remissions with normalization of peripheral blood values and the bone marrow in concert with a marked improvement in their immune defense towards infections. Likewise, IFN-alpha2 proved to be the first agent with the potential of inducing complete and sustained cytogenetic remissions with disappearance of Philadelphia chromosome in CML and—in addition—in some patients even the induction of major molecular remissions with a significant and sustained reduction of the BCR-ABL transcript in a subset of patients. These results were historical IFN milestones in the treatment of hematological malignancies (HCL and CML), which otherwise had a dismal prognosis with severe and often lethal atypical infections (HCL) or increasing genomic instability with terminal fatal leukemic transformation within a few years from the time of diagnosis in the large majority unless a bone marrow transplantation was an option (CML). Accordingly, IFN-alpha2 remained the best medical treatment of CML during the next decades until the targeted treatment with the tyrosine kinase inhibitor (TKI) imatinib mesylate substituted IFN-alpha2 about 20 years ago and later other TKIs (e.g., dasatinib and nilotonib) have entered as second-generation TKIs. As in CML, the mechanisms of action of IFN in patients with the Philadelphia-negative MPNs are likely multifactorial. In CML, IFN-alpha2 has been shown to restore the adhesion of CML primitive progenitor cells to marrow stroma, downregulate the expression of the BCR-ABL1, and activate several transcriptional factors that regulate cell proliferation, maturation, and apoptosis. In addition, very early in the IFN-era in CML, immune studies unraveled IFN-alpha to have very potent immune enhancing capacity, inducing recognition and elimination of CML cells by the immune system [11, 12]. Importantly, in 2009, a novel mechanism on hematopoietic stem cells (HSC) was described by Essers et al., implying induction of cell cycling in quiescent HSC and early progenitors by IFN-alpha2 [13]. One year later, they also showed that chronic administration of IFN-alpha2 depletes HSC, implying that “dormant” cancer stem cells may be susceptible to manipulation via an IFN-alpha2 induced “wake up call” with subsequent proliferation and “unmasking” of the malignant cells for the immune system by targeted treatment [14]. All these studies and the impact of IFN-alpha2 upon the immune system in CML [11, 12] created not only the platform for similar studies in patients with MPNs but also the platform for studies in CML patients on combination therapy with imatinib and IFN-alpha2 and later also studies on IFNs with other TKIs in CML [15–18]. Indeed, these studies have shown that such combination therapy is far more efficacious than singe-agent therapy based upon the fact that the modes of action and biological effects of TKIs and IFN-alpha2 are quite different. These lessons from the IFN-era in CML are of utmost importance, since so many similarities exist between the CML-IFN-landscape and the MPN-IFN-landscape in regard to highly important questions such as “Why to treat with IFN-alpha2?” and “When to treat with IFN-alpha2?”. Accordingly, several of the lessons in the CML-IFN era can be translated and used in the treatment of MPN patients today and in the future. All these questions will be addressed below.

Despite the very prominent “anti-cancer-effects” as described above, and despite initial studies displaying safety and efficacy of IFN-alpha2 in a large number of patients with the classical Philadelphia-negative MPNs—ET, PV, and MF (MPNs) [reviewed in 19–28], IFN-alpha2 disappeared in the dark and only in recent years the interest in using IFN-alpha2 in MPNs has been revived [19–50]. This renaissance of IFN-alpha2 in MPNs is mainly attributed to the increasing number of studies within the last 5–10 years, which have shown sustained complete hematological and major molecular remissions after long-term treatment with IFN-alpha2 [19, 20, 27–50], and have even been sustained up to 3 years after discontinuation of IFN-alpha2 [30, 31, 33, 41]. These highly encouraging and intriguing results envisage “minimal residual disease” (MRD) with normalization of peripheral blood cell values and normal bone marrow architecture to be new treatment objectives in MPNs [23–25, 51]. Importantly, they may also open a new horizon for patients with MPNs by promoting the next step towards cure by vaccination strategies as described elsewhere in this theme issue [52].

After a description of the history on IFN-alpha2 in MPNs, mechanisms of actions of IFN, and the novel concept of chronic inflammation as the driving force for clonal evolution in MPNs, we will focus on some controversial issues in MPNs and give our answers to key questions in MPNs—based upon decades of clinical experience with IFN-alpha2 in the treatment of MPNs and most recent novel observations. We will put in perspective the rationales for early treatment with IFN-alpha2-monotherapy in MPNs, for combination therapies, including JAK1–2 inhibitor (e.g., ruxolitinib), DNA-hypomethylators and statins, and the perspectives for such therapies to shape a new horizon with cure being an achievable goal together with vaccination strategies [51, 52].

## History of IFNs in MPNs

Already in 1985, Linkesch et al. from Austria described that IFN-alpha2 was able to control myeloproliferation in myeloproliferative diseases with severe thrombocytosis [53, 54]. Since then, several studies during the last 30 years have subsequently confirmed that IFN-alpha2 is also able to inhibit myeloproliferation in the Philadelphia-negative MPNs with a reduction or alleviation of the need of phlebotomies in PV, disappearance of pruritus, normalization of elevated leucocyte and platelet counts, and a reduction in spleen size [19–50, 55–59]. Although early studies in MPNs suggested that enhancement and modulation of immune cells might be involved in the mechanisms of action of IFN-alpha2 [58] and these aspects have been extensively studied in CML [11, 12], only recently immune cells and their functionality have been similarly studied during treatment with IFN-alpha2 [42, 49, 60–62]. Despite all these studies, IFN-alpha2 has not been the first drug of choice in the treatment of patients with MPNs, for many reasons but mainly because of a relatively high drop-out rate (about 20–40%) due to side effects [reviewed [19–27]. With the identification of the *JAK2*V617F-mutation in 2005 [63–66], reports on the potential of IFN-alpha2 to induce major molecular remissions in *JAK2*V617-positive patients [19, 26–50] and later on after the discovery of the *CALR*-mutations in 2013 [67–69], a reduction in the CALR-mutational load as well [39, 43], the interest in treatment of PV and related neoplasms with IFN-alpha2 has been revived as reviewed in several papers during the last 5–10 years [19–26, 55, 56]. Indeed, several studies have shown that long-term treatment with IFN-alpha2 in a subset of patients is accompanied by deep molecular remissions [30–34, 36–38, 41], which may be sustained even after discontinuation of IFN-alpha2 for up to 3 years [30, 31, 41]. These observations show that immune therapy with IFN-alpha2 is able to induce MRD (“operational cure”?) in subgroups of patients with MPNs.

## Mechanisms of action of IFN-alpha2

One of the major pathways by which IFN-alpha2 exerts its actions is the Janus-activated kinase/signal transducers and activators of transcription (STAT) signal pathway. The type I IFN-dependent signallying pathways are activated by both human type I IFN-a receptor chains 1 and 2 , their intracellular domains being associated with Janus-activated kinases, which accordingly are activated upon IFN-alpha2 binding to its receptors. Janus-activated kinases phosphorylate and activate STATs (pSTAT), which then translocate to the nucleus and activate gene expression [2–4, 6, 70].

The mechanisms of action of IFN-alpha2 have been ascribed to its antiproliferative, proapoptotic, antiangiogenic, and immunomodulatory effects [2–4, 70–90]. In addition, IFN-alpha2 has also been shown to downregulate telomerase reverse transcriptase and telomerase activity in both human malignant and non-malignant hematopoietic cells [91]. As interferon-alpha2 being a telomerase-inhibitor itself [91], it has been argued that the efficacy of another telomerase-inhibitor-imetelstat which recently has been investigated in ET and myelofibrosis patients [92–94] might actually be mediated through IFN-alpha2 [95] by binding of imetelstat to cell-surface receptors such as toll-like receptor 9 (TLR9) [95] with ensuing TLR9-induced production of type I interferons by plasmacytoid dendritic cells [96].

In most recent years, the impact of IFN-alpha2 upon the immune system has been studied extensively in patients with MPNs [42, 60–62, 97] and the studies by Riley et al. [60–62] have paved the way for vaccination studies in Danish MPN patients [98–102]. These studies of *JAK2*V617F-positive patients have shown marked changes in circulating immune cells with low levels of NK-cells, that are boosted during treatment with IFN-alpha2 [61, 62] and profoundly changing the NK-phenotype with a significant increase in the proportion of CD56bright NK cells and a decreasing CD56dim population. The findings in this study might indicate that IFN-alpha2 treatment skews the NK cell immunity towards a more immunostimulatory profile [61].

The frequency of circulating regulatory T cells—CD4 + CD25 + Foxp3+ T cells—(Tregs) was found to be significantly increased during IFN-alpha2 treatment in all patients [60, 62]. Myeloid dendritic cells (DCs) (mDCs) and plasmacytoid DCs (pDCs) displayed decreased frequencies during the course of treatment. On both mDCs and pDCs, HLA-ABC expression was upregulated, but decreased expression levels of HLA-DR were detected on mDCs. By whole-blood transcriptional profiling studies, we have previously described significant downregulation of HLA genes and speculated whether these findings might contribute to immune evasion of MPN cells [103] thereby reflecting immunoderegulation in MPNs [104] with deregulation of several immune genes [105–108] consequently giving rise to a defective tumor immune surveillance and an increased risk of second cancers, which has been demonstrated both before and after the MPN diagnosis [105–108]. Importantly, during treatment with IFN-alpha2, the downregulated HLA genes are upregulated, indicating that IFN-alpha2 is able to restore this defective component in the impaired immune surveillance [109]. It remains to be established whether long-term treatment may also decrease or eliminate the increased risk of second cancers in MPNs [106–108].

Interestingly, PD-L1 expression was reduced on mDC and increased on pDCs during treatment with IFN-alpha2 [62]. Importantly, we and others have most recently found PD-L1 upregulated in MPNs, this being yet another mechanism by which the malignant cells may evade the immune system in MPNs [102, 110]. Highly intriguing, Prestipino et al. show that the *JAK2*V617F induces the expression of PD-L1 through activation of STAT3, thereby likely mediating the immune escape in *JAK2*V617F-positive MPNs [110]. Since the *JAK2*V617F mutation is also a generator of reactive oxygen species (ROS) [111], it is relevant to consider whether the increased PD-L1 expression by *JAK2*V617F is further enhanced by inflammation.

The reasons for the consistent increase in circulating Tregs after institution of IFN-alpha2 [60, 62] might reflect IFN-alpha2-mediated mobilization of Tregs to the periphery [97]. If so, the migration of Tregs from the bone marrow to the periphery may decrease their immunosuppressive and tumor-promoting influence on the marrow microenvironment. An alternative interpretation might be that this expansion of Tregs reflects a counter-response to an overall activated immune system induced by IFN-alpha2 by unknown mechanisms and, thus, indeed represents a beneficial response to prevent auto-immunity as adverse effects to treatment [62].

In the above immune cell studies, no significant correlations were found between the changes in immune cells and hematological or molecular responses, which might be partly explained by a short interval of 9-month IFN-alpha2 treatment only. Similar studies after long-term treatment with IFN-alpha2 (> 12 months) are needed to assess whether the profound changes in circulating immune cells in the initial phase of IFN-alpha2 treatment are consistent and instrumental for the beneficial effects of long-term IFN-a2 treatment in some patients [62].

In the context of immune deregulation and defective immune surveillance as being potentially important mechanisms for clonal expansion in MPNs, it is intriguing to consider that the *JAK2*V617F mutation has been shown to generate the accumulation of ROS [111], thereby contributing to the chronic inflammatory state in MPNs (see below). In this regard, we have also by transcriptional profiling studies described a marked deregulation of oxidative and antioxidative stress genes [112], supporting the concept of chronic inflammation as the driving force for clonal evolution in MPNs. Most recently, our mathematical modeling studies have also delivered the proof of concept for MPNs as a human inflammation model for cancer development [113].

As previously alluded to, IFN-alpha2 has profound biologic effects on the MPN stem cells [13, 14, 114–116]. Pietras et al. elucidated the relationship between the proliferative and suppressive effects of IFN-alpha2 during acute versus chronic drug exposure [117]. These authors showed that the cell cycle entry due to acute exposure to IFN was but transient and that HSCs re-enter into quiescence during chronic IFN-alpha2 exposure [114]. Mullaly also demonstrated in a murine model of polycythemia vera that IFN-alpha2 depletes *JAK2*V617F myeloproliferative neoplasm-propagating stem cells [115]. Stein et al. have excellently described the biological rationales and use of IFN in MPNs [118].

## MPNs as inflammatory diseases

The MPNs are acquired stem cell diseases that include essential thrombocythemia (ET), polycythemia vera (PV), and primary myelofibrosis (PMF) [119, 120]. A long pre-diagnostic phase with abnormal hematological parameters usually precedes the final diagnosis [121, 122]. The MPNs have a low incidence but a prevalence comparable to lung cancer, since most MPN patients live for decades, although with a huge morbidity/comorbidity burden due to a high risk of cardio- and cerebrovascular complications, an increased risk of autoimmune and chronic inflammatory diseases [120–137], and an increased risk of second cancers (SCs) [105–107]. Even patients in the early cancer stages (ET and PV) exhibit shorter survival than the general population [119, 120]. Most recently, these blood cancers have been described as “a human inflammation model for cancer development” [124] reflecting chronic inflammation to be a major driving force for clonal evolution and disease progression [124–128] and accordingly contributing substantially to the symptom burden and an impaired quality of life (QoL) [129]. Chronic inflammation is the common link between highly prevalent diseases such as atherosclerosis, the metabolic syndrome, type II diabetes, and cancer [138–140]. Several of the signaling pathways activated in these diseases (e.g., the JAK-STAT pathway) are constitutively activated in MPNs due to driver mutations [63–69, 136]. Additional mutations are associated with an increased risk of leukemic transformation [69, 141]. Chronic inflammation is also involved in the huge inflammation-mediated disease burden [120–127, 130–134] very similar to that seen in patients with type II diabetes.

As previously noted, chronic inflammation has been suggested to be the driving force for clonal evolution, the development of premature atherosclerosis, and secondary cancers in MPNs [124–126], which accordingly have been described as “a human inflammation model” [124]. However, how chronic inflammation elicits MPN is a matter of intense investigation. By generating ROS, the *JAK2*V617 mutation is considered to be an important inflammatory driver [111]. In MPNs, the chronic inflammatory state per se with elevated levels of several inflammatory cytokines [126], deregulation of immune and inflammation genes [142–144], and/or oxidative stress and anti-oxidative defense genes [112] may all contribute to defective tumor immune surveillance, being most severely affected in the advanced myelofibrosis stage, where the deregulation of the above genes is most pronounced [112, 142–144]. In the context of the *JAK2*V617F mutation as a generator of ROS, it is most intriguing to note that the *JAK2*V617F mutation per se may actually modulate the T cell response by generating excessive ROS through an upregulation of Akt/phosphatidylinositol-3′-kinase, which in turn decreases the amount of the ROS-converting enzyme catalase [111]. Indeed, since ROS has been shown to be a potent inhibitor of T cell function [145, 146], it is tempting to speculate if the excessive ROS might attenuate the specific immune response against the *JAK2*V617F-clone. The implications of excessive ROS production in MPNs have previously been described and discussed [112, 147].

Most recently, another “inflammatory” mutation has been described in the background population—the *TET2*-mutation [148]. The Jaiswal paper brings several important pieces to the puzzle that might associate inflammation, atherosclerosis, and second cancer in MPNs. First, the *TET2* mutation gives rise to impaired resolution of inflammation by fostering the production of several inflammatory cytokines (e.g., IL-1beta and IL-6) [148] which are elevated in MPNs [126]. Second, *TET2* has been shown to exacerbate *JAK2*V617F-induced disease by eliciting prolonged leukocytosis and extramedullary hematopoiesis with splenomegaly and a shorter survival. It was concluded that the *TET2*-mutations might be a disease accelerator and disease initiator and sustainer in combination with *JAK2*V617F in MPNs [149]. Third, *TET2* loss leads to increased hematopoietic stem cell self-renewal and myeloid transformation [150], which might be explained by enhanced and sustained inflammation in the stem cell compartment [151] Fourth, *TET2* deficiency elicits monocytosis in mice and both the *TET2* mutation and monocytosis associate with inferior prognosis in MPNs [152–154]. Indeed, this association may explain the high cardiovascular morbidity and mortality in MPN patients with monocytosis and perhaps also the increased mortality associated with secondary cancers [105–108, 120–124, 131]. Accordingly, the *TET2* mutation may be yet another “inflammatory” mutation, which together with the *JAK2*V617F and *CALR* mutations may fuel the inflammatory drive, ultimately founding the soil for the development of overt MPN diseases from clonal hematopoiesis of indeterminate potential (CHIP) in the background population.

After the history on the journey of IFNs during the last 30 years, the mechanisms of action of IFN, and the novel concept of chronic inflammation as the driving force for clonal evolution in MPNs, we will in the following focus upon describing the rationales for IFN to be a successful story in the future treatment of MPNs and the perspectives for its use in MPNs. We do so by addressing some controversial issues in MPNs and provide our answers to some key questions.

## Some key questions on IFN-alpha2

### Does the efficacy of IFN-alpha2 reflect interference with a reactivated dormant virus—human endogenous retrovirus (HERV)?

Since IFN-alpha2 has highly potent antiviral activity, it is tempting to consider if the efficacy of IFN-alpha2 in MPNs reflects that IFN-alpha2 interferes with replication of a virus that is involved in the pathogenesis of MPNs. In this regard, particular attention has been payed to the potential role of human endogenous retrovirus (HERV), which has recently been revived as a potential causative factor for the development of MPNs [124]. Thus, the story on HERV being involved in MPN pathogenesis is not new. Indeed, HERV-K particles have been reported in megakaryocytes cultured from patients with ET [155, 156]. In the context of chronic inflammation as a potential trigger and driver of clonal evolution [124–126], it is intriguing to consider if the marked deregulation of inflammation and immune genes in MPNs [142–144]—several of these being deregulated in virus-induced malignancies as well—might be due to chronic inflammation elicited by a virus infection, e.g., reactivation of an endogenous retrovirus [124]. Thus, a chronic HERV infection of myeloid cells might account for activation of immune cells with deregulation of inflammation and immune genes. The immune attack with apoptosis of virus-infected cells might consequently elicit a sustained compensatory myeloproliferation of non-infected cells. However, ultimately, the immune system fails to clear the virus and from an early stage (ET) the disease progresses during the next 10–20 years in concert with a steady increase in bone marrow fibrosis, reflecting sustained reparative processes in an attempt to heal “the wound that won’t heal” [124, 157].

### Does interferon-alpha alter the frequency and functionality of immune cells in MPNs restoring a defective tumor immune surveillance?

As alluded to previously, IFN-alpha2 induces marked alterations in both the frequency and functionality of immune cells in MPNs [60–62]. Whole-blood gene expression profiling studies have unraveled massive deregulation of inflammation and immune genes with downregulation of several HLA-genes of importance for tumor immune surveillance [142–144]. Thus, immune deregulation in MPNs is well established [104]. Importantly, treatment with IFN-alpha2 is associated with upregulation of HLA genes [109] thereby improving the defective tumor immune surveillance. It has been speculated that the defective tumor immune surveillance might not only contribute to MPN disease progression through the biological continuum from the early cancer stages (ET and PV) to the advanced metastatic myelofibrosis stage but actually might also account for the increased risk of second cancers both before and after the MPN diagnosis [108]. The observation that discontinuation of IFN-alpha2 after long-term treatment (e.g., 5 years) may be followed by several years with normal cell counts and low-*JAK2*V617F burden (MRD) [30, 31, 33, 41] also support the concept that IFN-alpha2—by modulation and enhancement of the immune system and accordingly the defense against cancer development—is actually able to restore a defective tumor immune surveillance in MPNs with a sustained and powerful control of the malignant clone prohibiting clonal evolution. Studies are ongoing to elucidate if IFN-alpha treatment of MPNs may also reduce the increased risk of second cancers in MPNs as recently suggested [108] and most recently preliminarily described [158, 159].

### How does the mutational and cytogenetic landscape impact the efficacy of interferon-alpha2 in MPNs?

The mutational landscape in MPNs is complex and highly heterogeneous. Thus, in addition to the driver mutations—*JAK2*V617F, *CALR*, and *MPL*—several mutations outside the JAK-STAT pathway have been comprehensively described during the years [69, 141, 160]. Importantly, disease progression and clonal evolution in the biological continuum from the early cancer stages (ET/PV) along the path towards the advanced myelofibrosis stage have been closely linked to the development of additional subclonal mutations (*ASXL1*, *SRSF2*, *CBL*, *IDH1/IDH2*, *TP53*, and *SRSF2*), being independently associated with leukemic transformation and poor survival [69, 152, 160]. Thus, despite a low mutation rate, it has been shown that the presence of two or more somatic mutations significantly reduces overall survival and increases the risk for leukemic transformation in patients with MPNs [152].

Recent studies have suggested that mutations in the epigenetic modifiers—*TET2*, *DNMT3A*, *ASXL1*, *EZH2*, and *IDH1/2*—may lead to alterations in hematopoietic stem cell (HSC) function [150, 161–164]. Since IFN-alpha2 directly targets the malignant HCS [13, 14, 26], thereby potentially depleting and eliminating the disease-initiating HSC compartment [115], such alterations might negatively affect the response to IFN-alpha2. Indeed, in a small series of *JAK2*V617F patients, Kiladjian et al. showed that a subset had persistent *TET2*-positive clones during IFN-alpha2a treatment despite eradication of the *JAK2* mutations, indicating that IFN-alpha2 is able to reduce or eliminate the *JAK2*V617F mutant clone but not the *TET2* mutant clone [165]. These preliminary data might imply that patients with concurrent *JAK2*V617F and *TET2* mutations have a less favorable response to treatment with IFN-alpha2 taking into account that the *TET2* mutation—as the *JAK2*V617F mutation—is an “inflammatory mutation,” which gives rise to increased production of IL-6 and thereby an “inflammatory soil” in the bone marrow with potential impairment of IFN signaling and accordingly impaired clinical and molecular response to IFN-alpha2.

Highly interestingly, by serial sequencing of *TET2*, *ASXL1*, *EZH2*, *DNMT3A*, and *IDH1/2* in ET and PV patients treated with pegylated IFN-alpha2a, Quintas-Cardama et al. showed that the frequency of mutations in genes outside of *JAK2* was higher in patients failing to achieve a complete molecular remission (CMR) (56%) versus those achieving CMR (30%), although this difference did not reach statistical significance. Furthermore, patients not achieving CMR were more prone to acquire new mutations during therapy [36]. Of note, *TET2* mutations at therapy onset had a higher *JAK2*V617F mutant allele burden and a less significant reduction in *JAK2*V617F allele burden compared with *JAK2* mutant/*TET2* wild-type patients [36]. Surprisingly, in this study, *TET2* mutant alleles were shown to be eradicated by IFN-alpha2a in a subset of patents. However, all together, *TET2* mutant clones most commonly persisted during IFN-alpha2a treatment despite eradication of *JAK2*V617F mutant clones [36]. The authors speculated if the discovery that mutations in *TET2* [150, 162, 163, 166], *DNMT3A* [161], and *IDH1/2* [167] elicit an increased self-renewal might actually negatively influence the ability of IFN-alpha2 to reduce or eliminate mutant MPN disease initiating cells, which harbor these mutations and accordingly conferring acquired resistance to IFN-alpha2 [36]. The authors concluded that IFN-alpha2 induces CMR in a subset of PV or ET patients, and that the molecular signature may impact clinical and molecular responses to IFN-alpha2a [36]. Larger studies are needed to assess whether mutations in *TET2* and/or other genes that regulate the HSC compartment (such as *DNMT3a* and *IDH1/2*) result in persistence of malignant clones during IFN-alpha2 therapy and if their persistence indeed impact upon the prognosis of ET and PV patients being treated long-term with IFN-alpha2.

In regard to patients with early myelofibrosis, Silver and co-workers have most recently described the impact of the mutational landscape on the response to IFN-alpha2 in a phase 2 study of 30 patients with early myelofibrosis [117, 168], including their initial cohort of 17 patients [169–171]. The authors correlated response to IFN-alpha2 treatment with the mutation profile at the time of diagnosis, including both driver mutations (*JAK2*V617F, *CALR*, and *MPL*) and high risk mutations (HRMs), including *ASXL1*, *EZH2*, *SRSF2*, and *IDH1/2* [168]. Importantly, patients with these HRM did not respond to IFN-alpha2 therapy, irrespective of spleen size. Of note, the longest surviving patient who was in complete remission for more than 25 years had a molecular profile that included positive *CALR* and *TET2* mutation status. This observation is of utmost importance since it dictates that the *TET2* mutation may not consistently imply a poor response to IFN-alpha2 treatment [117, 168]. The findings by Silver et al. suggest that treatment with IFN-alpha2 in patients with early myelofibrosis may offer a survival benefit, putting in perspective the rationales for early therapeutic intervention with IFN-alpha2 in this patient group [22, 23, 51, 120] instead of “watchful waiting,” which is recommended in patients with low-risk MF at most MPN centers. The authors argue for early intervention with IFN-alpha2 before the development of the advanced myelofibrosis stage with large splenomegaly and bone marrow failure. At this stage of increasing genomic instability and subclone formation, IFN-alpha2 has only a minor impact, in part due to the presence of HRMs. The observations by Silver et al. substantiate “The Early IFN Intervention Concept” in MPNs [22–25, 51, 120], implying treatment with IFN-alpha2 to be initiated as early after the diagnosis as possible, when the tumor burden is at a minimum, because, at this stage, IFN-alpha2 is likely to have the optimal chance of inducing MRD as defined by normalization of the bone marrow and low *JAK2*V617F allele burden sustained even several years after discontinuation of IFN-alpha2 [22–25, 51, 120].

Several studies of smaller series of patients have documented cytogenetic remissions during treatment with IFN-alpha2 [reviewed in 24]. In recent years, larger studies, including the above study by Quintas-Cardama et al., have convincingly confirmed that long-term treatment with IFN-alpha2 may be followed by complete cytogenetic remissions [36, 37]. Thus, this highly important observation has also been confirmed by Gisslinger et al. using the new formulation of pegylated interferon alpha (peg-proline-IFNa-2b, AOP2014/P1101) [37]. In addition to high response rates being obtained on both hematologic and molecular levels, (the *JAK2*V617F mutational load) peg-proline-IFNa-2b treatment also led to cytogenetic remissions in a subset of their PV patients, even in those with complex cytogenetic findings at treatment onset [37]. In a previous study, Gisslinger et al. have reported that chromosomal aberrations emerged at the time of IFN-alpha2 resistance in a patient with primary myelofibrosis [172]. The impact of the mutational and cytogenetic landscape upon the immediate and long-term responses to IFN-alpha2 in MPNs remains to be definitely described in larger studies.

### How does the chronic inflammatory state in MPNs impact the efficacy of interferon-alpha2?

Chronic inflammation may impact the efficacy of IFN-alpha2 in MPNs. Thus, it has been shown that inflammatory signaling impedes the effect of IFN-alpha2 [173]. As previously alluded to, all effects of IFN-alpha2 on cells are elicited through interaction with the type I IFN receptor on the cell surface. This receptor consists of IFNAR1 and IFNAR2c chains. Among the potential mechanisms of refractoriness to IFN-alpha2 is downregulation of IFNAR1. Indeed, low levels of IFNAR1 correlate with poor response to IFN-alpha2 in patients with malignant melanoma [174]. Highly intriguing, Huang Fu et al. have shown that inflammatory cytokines interleukin 1-alpha (IL1-alpha) and tumor necrosis factor alpha (TNF-alpha)) stimulate IFNAR1 degradation and attenuate IFN-alpha signaling [173]. In patients with chronic hepatitis C, unresponsiveness to IFN-alpha is common, partly being explained by oxidative stress, impairing IFN-alpha signaling [175]. Since MPNs are associated with elevated levels of several inflammatory cytokines, including IL1-alpha and TNF-alpha, being produced by the malignant clone itself but also by the stroma cells in the bone marrow, and the highest levels have been reported in patients in the advanced myelofibrosis stage [126], these data also support the concept of early intervention with IFN-alpha2 when the inflammatory state is less pronounced. The fact that the effects of IFN-alpha are negatively impacted by inflammation may have several implications. First, one may speculate if smoking—exposing a huge systemic inflammatory load—may actually interfere with IFN signaling in MPN patients [176], implying either a weaker response to IFN-alpha2 or larger doses to be used to obtain adequate IFN responses in terms of inducing CHR. Second, agents with an anti-inflammatory potential in terms of lowering inflammatory cytokines, including IL1-alpha and TNF-alpha, might improve the IFN-alpha2 response. Indeed, the effects of IFN-alpha2 have most recently been shown to be enhanced by combination therapy with the JAK1–2 inhibitor, ruxolitinib, which is potently anti-inflammatory and immunosuppressive as well [177, 178]. Studies are ongoing to elucidate if statins, which have been suggested as potential useful agents in MPNs due to their anti-proliferative, anti-angiogenic, proapoptotic, and not least anti-inflammatory capabilities [179, 180], may also enhance the efficacy of IFN-alpha2 in MPNs. Taking into account that patients with MPNs have a 40% increased risk of second cancers [105], and statins have been shown to reduce cancer-associated mortality by 15% [181], their role in the treatment of MPNs certainly deserves to be investigated in the future [179, 180].

### Do we have predictors of IFN response in MPNs?

As earlier addressed, the mutational landscape may influence the response to IFN-alpha2. Highly interestingly, Andreassson et al. have recently shown that variation in IL28B genotype influences hematologic response in IFN-alpha2-treated MPN patients [50] similar to the response to IFN-alpha2 treatment of chronic hepatitis C, which has been shown to be strongly influenced by several related single nucleotide polymorphisms (SNP) in a region adjacent to the IL28B gene [182]. These observations are of utmost importance, and if confirmed in larger studies, they may help in identifying those patients who might benefit from IFN-alpha2 treatment.

## Rationales for treatment with IFN-alpha2 in MPNs

### Why to treat with IFN-alpha2?

As previously addressed, IFN-alpha2 is increasingly being recognized as the treatment of choice in the early disease stages (ET, PV) and in early myelofibrosis [19–50, 56] based upon safety and efficacy data on > 1000 patients being enrolled in single-arm studies during the last 30 years.

These studies have convincingly shown that complete hematological remissions (CHR) are achieved in the large majority with normalization of elevated cell counts within the first 6 months [19–50, 56] being accompanied by molecular remissions with a reduction in the *JAK2*V617F allele burden, in many patients already within the first few months and a subset of ET and PV patients achieving major molecular remissions after about 5-year IFN-alpha2 treatment. In a subset of patients, long-term treatment with IFN-alpha2 (approximately 5 years) is associated with normalization of the bone marrow, reflecting that IFN-alpha2 is a disease-modifying agent [22–25, 30, 31, 33, 41]. Since thrombocytosis is associated with an inferior prognosis in several cancers and MPNs are associated with an increased risk of second cancers [105, 106] that also have an inferior prognosis as compared to the background population [106], it seems highly relevant to normalize elevated platelet counts in patients [108]. Importantly, elevated platelet counts may attribute to the inferior survival of second cancers in MPNs, since platelets enhance cancer invasiveness in solid tumors and accordingly their metastatic potential [183]. In addition, platelets surround tumor cells during their journey to metastatic sites, thereby protecting them from being attacked and killed by NK-cells. In this perspective, it seems most rational to normalize elevated platelet counts by IFN-alpha2, which concomitantly strongly enhance and boost the number and functionality of several immune cells, including NK-cells. These important aspects have recently been described as “The Platelet-Cancer –Loop in MPNs” [183]. Another heavy-weight rationale includes the fact that both leukocytes and platelets are deeply involved in the atherosclerotic process and leukocytosis is a risk factor for thrombosis—both in the background population and in patients with MPNs [125]. Accordingly, sustained leukocytosis and thrombocytosis are likely key players in the development of premature atherosclerosis in MPNs, being also substantiated by the association between the occurrence of the *JAK2*V617F-mutation and ischemic heart disease in a large epidemiological study [184]. In this study, the *JAK2*V617-mutation was also linked to the emergence of second cancers [184], raising the possibility that the *JAK2*V617F mutation actually is a “tumor promoter” not only eliciting genomic instability in blood cells but also increasing the risk of other cancers—perhaps by generating ROS and chronic inflammation in several organs other than the bone marrow compartment [108].

### When to start treatment with IFN-alpha2?

All untreated cancers progress from an early stage to the advanced metastatic stage due to increasing genomic instability, subclone formation, and ultimately metastasis. As cancers, the MPNs are no exception to this general rule on cancer biology. Accordingly, institution of IFN at the earliest time point possible in MPNs may offer the best chance of a successful outcome [22–25]. The “Early IFN Intervention Concept” is based upon Danish studies, which have demonstrated that long-term treatment with IFN may induce a state of MRD as defined by deep molecular remissions (< 1% mutated *JAK2*V617F alleles) in concert with a normalization of the bone marrow—even being sustained in a subset of patients after discontinuation of IFN for several years [30, 31, 33, 41]. Since chronic inflammation may be a highly important driving force for clonal evolution in MPNs, combination therapy with the JAK1–2 inhibitor ruxolitinib and IFN (COMBI) has recently been suggested to be a rational treatment modality [51] being based upon the first clinical observation in a Danish PV-patient treated with COMBI [177] and the highly encouraging results in the Danish COMBI trial [178].

### Side effects of IFN-alpha2

IFN-alpha2 treatment is associated with side effects that account for drop-out rates of about 20–30% in most studies, even when using low-dose pegylated IFN-alpha2 [19–48]. Many patients only experience the initial flu-like symptoms and afterwards they tolerate Peg-IFN-alpha2 exceedingly well. However, in some patients, chronic fatigue and/or musculoskeletal pain may persist, ultimately necessitating withdrawal of the treatment. A minority of patients develop depression which necessitates pausing or discontinuation of IFN-alpha2. In patients with previous or present psychiatric disease, IFN-alpha2 should be administered cautiously. Some patients may develop symptoms and signs of autoimmune disease. Thyroid dysfunction—thyroiditis with ensuing hypothyroidism—may develop in a subset of patients and accordingly it is recommended to test thyroid function before and during treatment. Other rare autoimmune diseases include polyarthritis, dermatomyositis, immune hemolytic anemia, immune thrombocytopenia, and glomerulonephritis.

In previous studies in patients with CML and in patients with malignant melanoma, the development of autoimmune phenomena/diseases during IFN-alpha2 treatment has been linked to an enhanced anti-leukemia or antitumor effect reflecting a very efficient immune attack on the malignant cells.

Whether similar associations exist in patients with MPNs has never been investigated. A comprehensive description of side effects to IFN-alpha2 in patients with MPNs has been given in several reviews during recent years [22–26].

## Conclusion and perspectives

The MPNs are inflammatory cancers, in which the malignant clone per se generates inflammatory products that in a self-perpetuating vicious circle sustain the inflammatory drive and accordingly disease progression in the biological continuum from the early cancer stages (ET/PV) to the advanced “burnt-out” myelofibrosis stage and imminent leukemic transformation [120–128]. During this evolution, additional mutations, other than the driver mutations, emerge. The MPNs are associated with several “inflammatory” co-morbidities, including an increased risk of second cancers [105–108], which are likely due to a defective tumor immune surveillance system being partly attributed to the chronic inflammatory state [108].

The cornerstone treatment of MPNs in the future is foreseen to be IFN-alpha2, which as monotherapy in several studies during the last three decades has demonstrated safety and efficacy and as the only agent within MPNs is able to induce MRD and accordingly being disease modifying [30, 31, 33, 41]. Thus, recently, the apparent disease-modifying potential of IFN-alpha2 in PV and ET as evidenced by the progressive reduction of the *JAK2*V617 tumor burden during prolonged therapy has elicited renewed efforts to evaluate its clinical efficacy as front-line therapy for early stage disease in terms of reducing thrombo-hemorrhagic events, normalization of biochemical, hematologic, and molecular variables, and, ultimately, altering the natural history of these diseases.

The perspectives for the future treatment of MPNs with the goal of inducing MRD and hopefully cure in a subset of MPN patients are combination therapies, in which IFN-alpha2—primarily and directly targeting the malignant clone [26]—is being combined with agents targeting the concurrent inflammatory state (JAK1–2 inhibitors and statins), that are driving clonal expansion and disease progression [124–126]. The rationales for these combinations have been thoroughly described and discussed in most recent reviews [51, 124–128], and preliminary results from the first Danish studies are indeed very promising [177, 178]. In patients in the accelerated phase towards leukemic transformation and in patients having transformed to acute myeloid leukemia, the prognosis is dismal [185]. However, even in these stages, IFN-alpha2 may be an option [186] with the potential as monotherapy to revert imminent or overt leukemic transformation [186]. Importantly, recent studies have shown that monotherapy with the DNA-hypomethylator azacytidine [187] may be efficacious in these patients, and combination therapy with a DNA-hypomethylator and ruxolitinib may be even more efficacious [188]. Based upon the above studies of monotherapy with IFN-alpha2 and combination therapy with DNA-hypomethylating agents and ruxolitinib in patients towards or with leukemic transformation, it is intriguing to consider if “triple therapy” (IFN-alpha2 + DNA-hypomethylator + ruxolitinib) may be even more efficacious. The rationales for this “triple therapy” are several. First, such a combination directly targets the malignant clone (IFN-alpha2 + DNA-methylator) and dampens the fire—the inflammation—that fuels the malignant clone. Second, as noted above, hypomethylators have shown efficacy as monotherapy in MPN patients in the accelerated phase [187] and combination therapy (aza and ruxolitinib) seems even more efficacious [188]. Third, Aza stimulates the expression of retroviral proteins, and this expression of retroviral proteins activates immune signaling through the viral defense pathway causing a type I interferon response and apoptosis [189]. Fourth, the type I interferon response is accompanied by upregulation of hypermethylated endogenous retrovirus (ERV) genes and ERV overexpression which activates the response [190]. Fifth, by stimulating the expression of retrovirus (virus mimicry) [190], aza may render MPN cells more immunogenic and thus more susceptible to attack by immune cells. Sixth, by enhancing immune cell function, IFN may—in combination with aza—further accelerate MPN cell killing.

Most recently, the *JAK2*V617F and the *CALR* mutations, found in > 90% of patients, were shown to be highly immunogenic neo-antigens [98–101]. Additionally, patients with MPN display frequent and strong T cell responses against the immunoregulatory proteins programmed death ligand-1 (PD-L1) and arginase-1 [102, 191]. Accordingly, peptide vaccination with either *JAK2*-mutant or *CALR*-mutant epitopes in combination with vaccination against PD-L1 and/or arginase-1 may be a new and potentially curable treatment modality for MPN [98–101] as also reviewed by Holmström and Hassselbalch elsewhere in this theme issue [52].

By early detection of MPNs at the earliest time point in target populations in combination with early intervention with IFN and in subsets of patients COMBI, it is envisaged that MRD may be induced in a substantial proportion of patients along the path towards ultimate cure being obtained by novel vaccination strategies. The IFN story in MPNs will never end.
